# A Phase 3, Randomized, Double-Blind Comparison of Analgesic Efficacy and Tolerability of Q8003 vs Oxycodone or Morphine for Moderate-to-Severe Postoperative Pain Following Bunionectomy Surgery

**DOI:** 10.1111/pme.12167

**Published:** 2013-06-26

**Authors:** Patricia Richards, Dennis Riff, Robin Kelen, Warren Stern

**Affiliations:** * QRxPharma Inc.Bedminster, New Jersey; † Advanced Clinical Research InstituteAnaheim, California, USA

**Keywords:** Acute Pain, Opioid, Q8003, Morphine, Oxycodone, Postoperative Pain, Opioid-Related Side Effects

## Abstract

**Objective:**

Compare the efficacy and tolerability of the dual-opioid, Q8003® (morphine/oxycodone combination) 12 mg/8 mg to morphine 12 mg or oxycodone 8 mg in subjects following bunionectomy surgery.

**Design:**

This was a randomized, double-blind study.

**Setting:**

Hospitalized patients.

**Patients:**

Healthy men or women aged ≥18 years with moderate or severe pain (score ≥2 on a 4-point Likert scale) and ≥4 on the 11-point numerical pain rating scale following surgery.

**Interventions:**

Study medication was initiated after surgery and was given for 48 hours.

**Outcomes:**

The primary efficacy variable was mean sum of the pain intensity difference (SPID) scores from the postsurgical baseline.

**Results:**

Five hundred twenty-two subjects were randomized; 31 (5.9%) discontinued, including 19 (3.6%) for adverse events. The mean total morphine equivalent dose (MED) was 182.7 mg from Q8003 12 mg/8 mg, 92.4 mg for morphine 12 mg, and 92.1 mg for oxycodone 8 mg. SPID from baseline over 24 hours and SPID from baseline over 48 hours were significantly (*P* < 0.02) higher for Q8003 12 mg/8 mg vs morphine 12 mg or oxycodone 8 mg. Significantly (*P* < 0.015) fewer subjects in the Q8003 group required ibuprofen rescue medication, used lower doses of rescue medication, and had a longer median time to first use of rescue medication. Oxygen desaturation <90% occurred in 5.3% with Q8003, 2.8% with morphine 12 mg, and 2.3% with oxycodone 8 mg, and the cumulative median dose at first desaturation was twofold greater with Q8003.

**Conclusion:**

Q8003 provided superior efficacy to its individual components at twice the MED with only a modest increase in the incidence of adverse events.

## Introduction

Opioid medication is the mainstay of pain management following most surgical procedures or significant injury. Immediate release formulations of morphine, oxycodone, other opioids, and combination products containing nonsteroidal anti-inflammatory drugs or acetaminophen are most commonly administered for moderate-to-severe acute pain 1. In fact, the combinations of hydrocodone plus acetaminophen or oxycodone plus acetaminophen are among the most widely used drugs of any therapeutic class.

Single entity opioids or these combination products, while certainly effective, can have worrisome side effects. Over 90% of patients report at least one adverse event (AE) from opioid treatment of acute pain 2. The most common AEs are gastrointestinal (GI), central nervous system effects, respiratory, pruritus, and urinary retention, resulting in lower than optimal doses of opioids being used in 50–60% of patients 3. Over one-third of acute pain patients are bothered by GI AEs, and >50% indicated that a reduction in AEs was the most important unmet need for pain medications 2. Opioid-associated GI side effects place a substantial burden on the patient by impairing well-being, functional ability, and work productivity, and on costs associated with their health care 4. In addition, the use of acetaminophen as a component of a combination product with hydrocodone or oxycodone, for example, has recently come under Food and Drug Administration (FDA) scrutiny for safety reasons related to potential liver toxicity associated with the acetaminophen component, especially when patients concurrently ingest acetaminophen from cough-cold or other analgesic products. A recent FDA ruling that will take effect in January 2014 limits the acetaminophen dosage in opioid combination products to 325 mg, thereby relying more on the opioid component (up to about 15 mg morphine equivalent dose [MED] per dose) 5. Thus, there is a need for additional opioid products of sufficient analgesic strength with good tolerability to serve as an alternative to single entity opioids or acetaminophen-containing opioid combinations.

While some may consider morphine and oxycodone to be essentially the same drugs from a clinical perspective, these two opioids differ in a number of potentially important ways that may give rise to clinical advantages when administered in a single combination product. In respect to opioid receptors, it is well established that mu, kappa, and delta receptors are present in the brain and spinal cord, and that these receptors have many subtypes 6. Pasternak has shown that the binding of a given opioid to an array of these subreceptors differs from one person to the next, as well as from one opioid to the next 6. In the case of mu receptors, at least 10 receptor subtypes have been identified, and the binding of morphine to these subtypes differs between individual 6. Further, while morphine is generally recognized to be a pure mu agonist, pharmacological evidence exists that oxycodone has both mu and kappa-2 receptor binding properties 7 and that when morphine and oxycodone are coadministered, both the analgesic properties and sedating effects of the combination is markedly different than that of either drug given alone 8. Other researchers have shown that combinations of other opioids also produce unexpected synergy in analgesic effects 9. See Pasternak 10 for a review of novel pharmacological effects of opioids when they are given concurrently, including changes that may lead to reduced adverse effects. The incomplete cross-tolerance between morphine and oxycodone seen clinically is also an evidence that these are distinct opioids, which differ in ways other than relative bioavailability, metabolic pathway, or relative potency.

Currently, no product containing morphine plus oxycodone or other opioid-opioid combinations is marketed in the United States or elsewhere. Controlled clinical studies of the coadministration of morphine plus oxycodone demonstrate excellent analgesia with a lower incidence or less intense AEs, especially nausea and vomiting, compared with equi-analgesic doses of other opioids 11–16. Other studies indicate there may be a marked reduction in the risk of respiratory impairment with morphine plus oxycodone compared with equi-analgesic doses of morphine or of oxycodone 17, 18.

In order to obtain FDA approval for a combination drug product, evidence from an adequate, well-controlled trial is needed to demonstrate that the combination product is superior to its individual components (FDA Draft Guidance for Industry on Codevelopment of Two or More Investigational Drugs for Use in Combination, 2010). This study was conducted to satisfy FDA requirements for the efficacy of a combination product. The objective of this phase 3, randomized, double-blind study was to compare the efficacy, safety, and tolerability of the dual-opioid, Q8003® (capsule of morphine plus oxycodone; DSM Pharmaceuticals Inc., Greenville, NC, USA) 12 mg/8 mg vs its monotherapy components, morphine 12 mg or oxycodone 8 mg in subjects with moderate-to-severe pain following unilateral bunionectomy surgery.

## Methods

### Study Design

This was a randomized, double-blind, parallel-treatment, three-arm, fixed-dose factorial design study conducted at six clinical sites in the United States of Q8003 12 mg/8 mg vs morphine 12 mg and oxycodone 8 mg for the treatment of moderate-to-severe postoperative pain following bunionectomy. The study protocol and informed consent form were approved by Aspire IRB, La Mesa, California, USA, and Copernicus Group IRB, Research Triangle Park, North Carolina, USA, prior to initiation of the study. All subjects or their representatives provided written informed consent prior to study participation. This study was conducted in accordance with the principles of the Declaration of Helsinki and met Good Clinical Practices and applicable regulatory requirements.

Eligible subjects were randomly allocated in equal proportion (1:1:1) to Q8003 12 mg/8 mg, morphine 12 mg, or oxycodone 8 mg. Randomization was stratification by site using a centralized interactive voice response system to allocate treatment assignment. Morphine-equivalent doses were calculated using a conversion multiplier of 1.0 for mg dose of oxycodone to 1.5 mg dose of morphine. Study drug was dosed every 6 hours, with the last dose of study medication given at 42 hours following the start of dosing.

### Subject Selection

Healthy men or women aged ≥18 years were eligible for this study if they underwent uncomplicated, unilateral, first metatarsal bunionectomy under regional anesthesia and met criteria for American Society of Anesthesiologists Class I–III. Women were nonpregnant, nonlactating, and practicing an acceptable method of birth control (double-barrier, hormonal contraceptives, or abstinence) or were surgically sterile or postmenopausal. A pregnancy test was obtained at screening and prior to surgery. Subjects were required to have moderate or severe pain (score ≥2 on a 4-point Likert scale and ≥4 on the 11-point numerical pain rating scale [NPRS] where 0 = no pain and 10 = worst imaginable pain, have a pulse oximetry measurement of at least 95% oxygen saturation, and have a respiratory rate of at least 12 breaths/min).

A subject was excluded for any acute or chronic medical condition that could interfere with the conduct of the study, current use of a nonopioid analgesic at doses that, in the opinion of the investigator, would interfere with evaluations of the study medications; history of hypersensitivity or poor tolerance to ibuprofen or to short-term opiate use (including tramadol); use of opiates (including tramadol) continuously for more than 10 days in the past year; current use of any medications that were not at a stable dose for at least 2 months prior to date of surgery; history of sleep apnea or receiving any form of treatment for sleep apnea at the time of enrollment; use of systemic or intra-articular corticosteroids within 14 days prior to surgery; use of any drug or alcohol that could interfere with the conduct of the study; and body mass index (BMI) ≥35 kg/m^2^.

### Study Procedures

**Figure 1:**
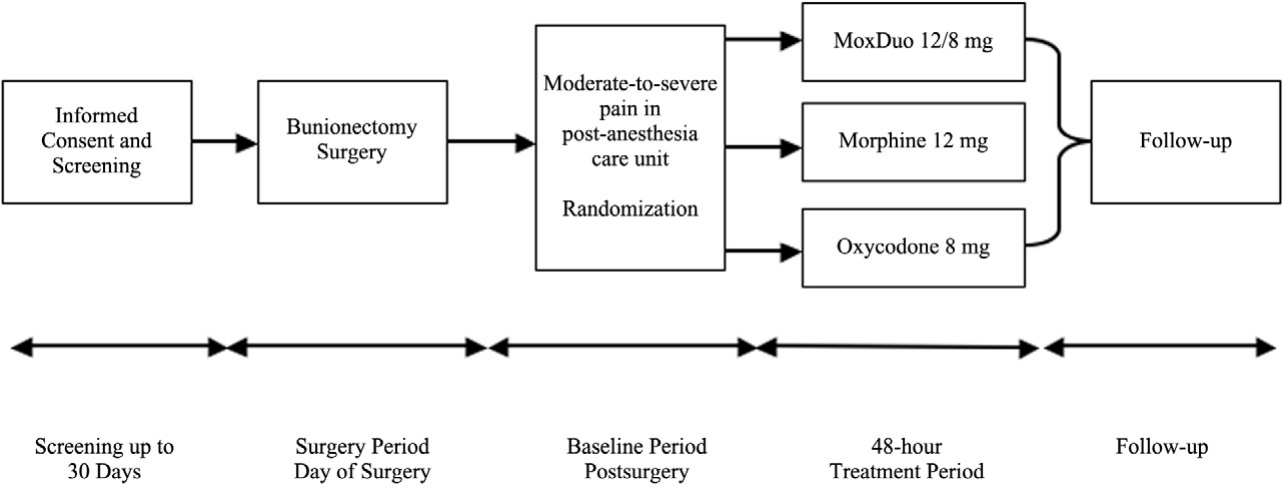
Study flowchart.

Subjects were screened for up to 30 days prior to surgery (Figure 1). Within 6 hours postsurgery, subjects whose pain intensity met the inclusion criteria were randomized to double-blind treatment with study medication every 6 hours for 48 hours. Subjects remained at the study center during the 48-hour treatment period during which time efficacy and safety assessments were conducted. During the double-blind treatment period, administration of study medication continued unless the subject experienced a safety issue: oxygen saturation <90% assessed using continuous pulse oximetry, respiratory rate <10 breaths/min, pulse rate outside the 50–100 beats/min range, or systolic and diastolic blood pressure outside of specified parameters.

Ibuprofen 400 mg every 4–6 hours as needed not to exceed 3,200 mg/day was provided as supplemental analgesia. Subjects were discouraged from requesting ibuprofen within an hour after the first dose of study medication. Final safety data were collected from study subjects by telephone, mail, or email within 48 hours after they were discharged from the study unit.

### Study Assessments

Pain intensity (NPRS), vital signs, and oxygen saturation were assessed just prior to the first dose and at 15, 30, 45, and 60 minutes, and 2, 3, 4, 6, and 48 hours (or at early discontinuation) after the first dose of study medication. Pain intensity was rated by subjects just prior to and 1 hour after each dose of study medication, and just prior to administration of supplemental analgesia (if needed) throughout the 48-hour treatment period. Pain relief (5-point pain relief scale) was assessed at the same time points as pain intensity with the exception of the baseline determination.

A responder at 24 and at 48 hours of treatment was defined as subjects who either rated 3 (good) or 4 (excellent) using the Subject Global Assessment scale and/or had at least a 30% improvement in the NPRS score from baseline to 24 or 48 hours. The number and proportion of subjects using rescue medication, the dose, and the time to first use were recorded.

AEs were recorded by study staff throughout the study, including time of onset, intensity, duration, causality, action taken, and outcome. The definitions for the severity of AEs were as follows: “awareness of sign or symptom, but easily tolerated” (mild), “discomfort sufficient to cause interference with normal activities” (moderate), or “incapacitating, with inability to perform normal activities” (severe). Other safety assessments included physical examinations, electrocardiogram, vital signs (i.e., pulse, blood pressure, and respiratory rate), and laboratory testing at different study time points.

### Statistical Analysis

Efficacy analysis was carried out using the intent-to-treat population, defined as all subjects treated with at least 1 dose of study medication and who reported a baseline pain intensity score. The safety population included all subjects who received at least one dose of study medication. Data from a previous study 16 were used to determine sample size for this study. The observed minimum treatment group difference for the sum of the pain intensity differences (SPID) from baseline over 48 hours (SPID_48_) for Q8003 12 mg/8 mg vs oxycodone 8 mg was 38.1 units 16. This study was designed to detect a treatment difference of 32 units, which is analogous to a two-thirds unit decrease in pain per hour over 48 hours. The selection of 32 units was chosen to provide a more conservative estimate of the treatment difference. Assuming a common standard deviation (SD) of 106 and a power of 80%, the sample size in each of the treatment arms would be 174 or 522 subjects total.

Descriptive summaries for subject demographics, clinical characteristics, clinical laboratory values, and AEs were presented by mean, SD, and percentage.

The primary end point of this study was SPID_48_ after the first dose of study medication. The pain intensity difference (PID) was calculated for all time points when pain intensity was measured. The time-weighted SPID was calculated for 6, 24, and 48 hours after the first dose of study medication. An analysis of covariance (ANCOVA) model was used to compare treatment effects between groups considering center, baseline pain score, age, gender, and BMI. No adjustments for multiple comparisons were made in this study. Least squares (LS) mean values and standard error were used for efficacy end-point summaries. A logistic regression method was used to compare proportion of responders and subjects who needed rescue medication, and a chi-square test was used for group comparisons. Log-rank test was used to compare time to first use of rescue medication between groups. All statistical tests were two-sided, and significance was based on *P* < 0.05.

Missing efficacy data were imputed for primary and secondary end points. For instances in which supplemental analgesic medication was taken during the treatment period, pain intensity and pain relief scores were imputed using the last observation carried forward (LOCF) method. If a subject withdrew from the study prior to the end of the treatment period due to an AE, pain intensity and pain relief scores were imputed using the baseline observation carried forward method. If an AE was not the reason for withdrawal, pain intensity, and pain relief scores were imputed using the LOCF method. In the event that a subject completed the treatment period but did not have pain intensity and/or pain relief assessments recorded at 48 hours after the first dose of study medication (±1 hour), pain intensity and/or pain relief values at the 48-hour time point were imputed using the LOCF method.

## Results

Following surgery and the onset of moderate or severe pain, 522 subjects were randomized to study treatment, and 491 (94.1%) completed the study (Table 1). Thirty-one (5.9%) subjects discontinued the study, including 19 (3.6%) for AEs. The rate of discontinuation across all reasons was comparable across the treatment arms (range 5.1–7.0%). At postsurgical baseline just prior to randomization, more patients in the Q8003 group (52%) had severe pain (NPRS ≥ 7) than in the morphine (38%) or oxycodone (46%) groups (Table 2). The mean total MED was 182.7 mg from Q8003 12 mg/8 mg, 92.4 mg for morphine 12 mg, and 92.1 mg for oxycodone 8 mg.

**Table 1:** Disposition of study subjects

	Number (%) of Patients		
	
	Q8003 12 mg/8 mg	Morphine 12 mg	Oxycodone 8 mg
Randomized	171	176	175
Completed	159 (93.0)	167 (94.9)	165 (94.3)
Discontinued	12 (7.0)	9 (5.1)	10 (5.7)
Reason for withdrawal			
Withdrew consent	2 (1.2)	2 (1.1)	1 (0.6)
Adverse event	8 (4.7)	4 (2.3)	7 (4.0)
Lack of efficacy	2 (1.2)	1 (0.6)	1 (0.6)
Protocol violations	0	2 (1.1)	1 (0.6)

**Table 2:** Baseline demographic and clinical characteristics

	Q8003 12 mg/8 mg (N = 171)	Morphine 12 mg (N = 176)	Oxycodone 8 mg (N = 175)
Sex, N (%)			
Male	26 (15.2)	25 (14.2)	28 (16.0)
Female	145 (84.8)	151 (85.8)	147 (84.0)
Mean (SD) Age, year	44.3 ± 12.5	42.6 ± 14.0	44.6 ± 13.1
Mean (SD) BMI (kg/m^2^)	25.7 ± 4.1	25.4 ± 4.3	26.4 ± 4.4
Race/ethnicity, N (%)			
White	107 (62.6)	102 (58.0)	99 (56.6)
Black	32 (20.5)	33 (18.8)	49 (28.0)
Asian or Indian	10 (5.8)	9 (5.1)	7 (4.0)
Other	19 (11.1)	32 (18.1)	20 (11.4)
Hispanic or Latino	42 (24.6)	45 (25.6)	34 (19.4)
Baseline NPRS score ≥ 7, N (%)	89 (52.0)	66 (37.5)	81 (46.3)

BMI = body mass index; NPRS = numerical pain rating scale; SD = standard deviation.

### Efficacy

**Figure 2:**
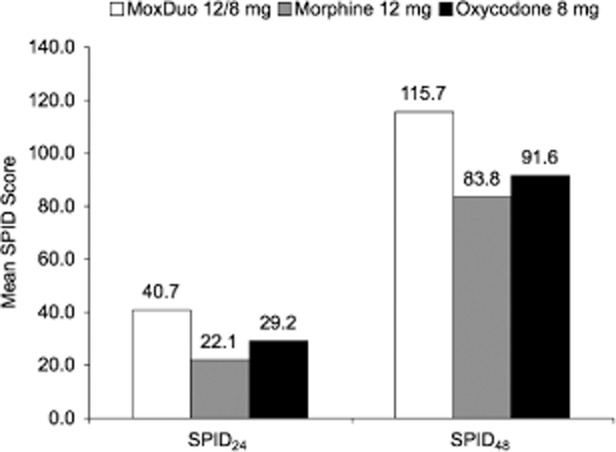
Mean sum of the pain intensity difference (SPID) from baseline over 24 hours (SPID_24_) and SPID from baseline over 48 hours (SPID_48_) values for Q8003 12 mg/8 mg, morphine 12 mg, and oxycodone 8 mg. For SPID_24_, Q8003 was significantly better than morphine 12 mg (*P* = 0.003) and oxycodone 8 mg (*P* = 0.027). For SPID_48_, Q8003 was significantly better than morphine 12 mg (*P* = 0.020) and oxycodone 8 mg (*P* = 0.019) using the analysis of covariance model.

Mean SPID from baseline over 24 hours (SPID_24_) and SPID_48_ values were significantly (*P* < 0.02) higher for Q8003 12 mg/8 mg vs morphine 12 mg or oxycodone 8 mg (Figure 2). LS mean differences between treatments were significant (*P* < 0.05) for Q8003 12 mg/8 mg vs the comparators for both SPID_24_ and SPID_48_. LS mean differences also were significantly greater with Q8003 12 mg/8 mg than with morphine 12 mg (*P* < 0.001) but not oxycodone 8 mg for SPID_6_.

**Figure 3:**
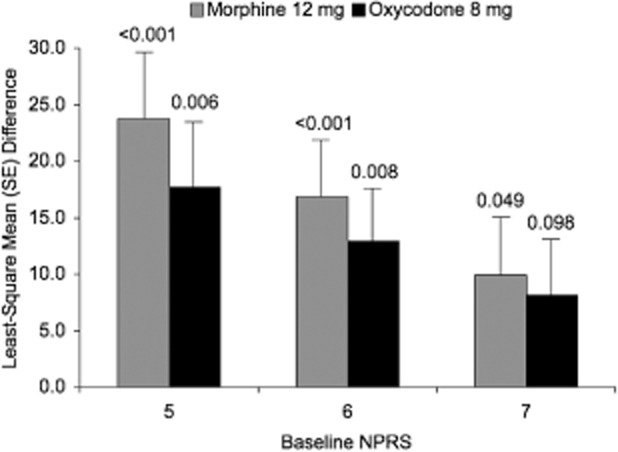
Sum of the pain intensity difference from baseline over 24 hours by baseline pain intensity score of 5, 6, or 7 on the numerical pain rating scale (NPRS) was used to compare treatment effects and the analysis of covariance included treatment, site, gender, age, body mass index, treatment baseline, and baseline score as fixed-effects.

The magnitude of reductions in pain using the SPID_24_ was evaluated for subjects with baseline pain intensity scores of 5, 6, or 7 on the NPRS (Figure 3). SPID_24_ was significantly improved (*P* < 0.05 to *P* < 0.001) with Q8003 12 mg/8 mg compared with morphine 12 mg at all levels of baseline pain and was significantly (*P* = 0.006 and *P* = 0.008) improved relative to oxycodone 8 mg at baseline pain intensity of 5 or 6, despite the marked reduction in sample sizes and statistical power associated with these subanalyses.

From 0 to 24 hours, the proportion of responders was 64.9% with Q8003 vs 54.5% with morphine 12 mg and 52.0% with oxycodone 8 mg. This difference was significant with Q8003 12 mg/8 mg vs oxycodone 8 mg (*P* = 0.013, logistic regression model) and marginally nonsignificant vs morphine 12 mg (*P* = 0.068).

**Figure 4:**
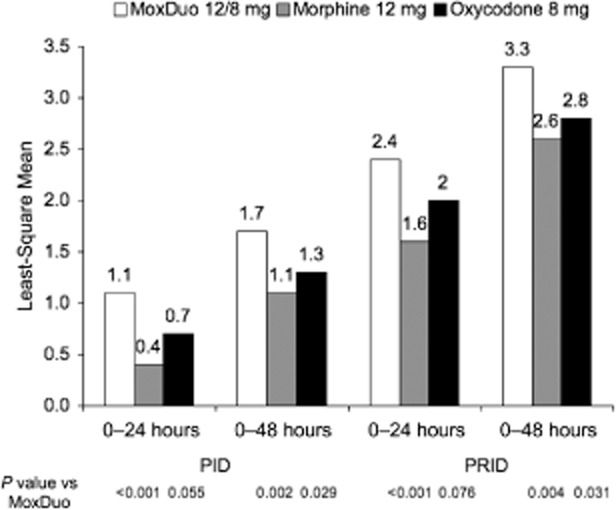
Least-square mean pain intensity difference (PID) and pain relief difference (PRID) scores at 0–24 and 0–48 hours for each treatment group. *P* values are from an analysis of covariance model for Q8003 vs morphine or oxycodone.

PID and pain relief difference (PRID), the ANCOVA model included treatment, site, and baseline score as fixed effects, and the mean of all scores was used for each time point. Subjects in the Q8003 12 mg/8 mg group had a significantly higher PID score than subjects in the morphine 12 mg group from 0 to 24 and from 0 to 48 hours (*P* < 0.001 and *P* = 0.002, respectively) and subjects in the oxycodone 8 mg group from 0–48 hours (*P* = 0.029) (Figure 4). Subjects in the Q8003 12 mg/8 mg group had a significantly higher PRID score than subjects in the morphine 12 mg group from 0 to 24 and from 0 to 48 hours (*P* < 0.001 and *P* = 0.004, respectively) and subjects in the oxycodone 8 mg group from 0 to 48 hours (*P* = 0.031).

**Figure 5:**
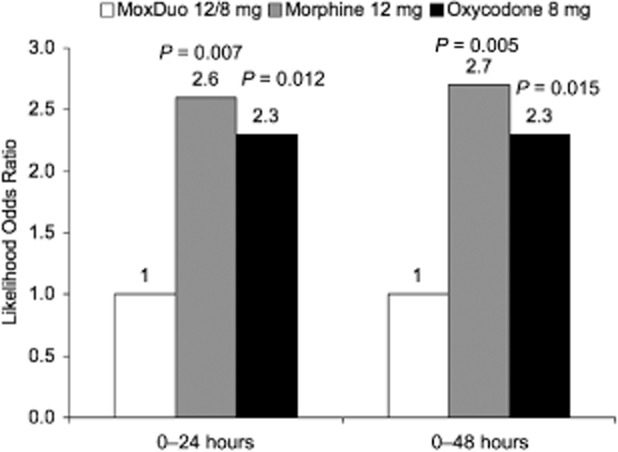
Cox regression analysis of the need for rescue medication at 0–24 and 0–48 hours. *P* values represent comparisons of Q8003 vs morphine or oxycodone using the chi-square test.

**Figure 6:**
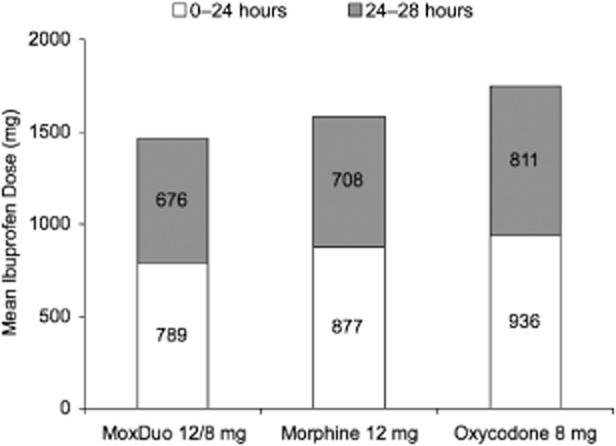
Mean total dose of ibuprofen used as rescue medication.

The proportion of subjects who received rescue medication at any time during the study was smaller with Q8003 12 mg/8 mg (82.5%) compared with morphine 12 mg (92.6%) and oxycodone 8 mg (91.4%). Cox regression analysis of the need for rescue medication was significantly lower (*P* < 0.015 to 0.005, chi-square) with Q8003 12 mg/8 mg than with morphine 12 mg or oxycodone 8 mg (Figure 5). The mean dose of ibuprofen was lower with Q8003 12 mg/8 mg at 0–24 and 24–48 hours vs morphine 12 mg and oxycodone 8 mg (Figure 6). The median time to first use of rescue medication ranged from 2.01 to 2.22 hours. The median time to first rescue medication was significantly longer with Q8003 12 mg/8 mg compared with morphine 12 mg or oxycodone 8 mg (*P* < 0.001 and *P* = 0.008, respectively, log-rank test).

### Tolerability

While treatment-emergent AEs (TEAEs) occurred in the majority of subjects and at a higher rate with Q8003 (Table 3), the incidence of severe TEAEs and discontinuation for AEs was comparable between treatment groups. No deaths occurred, and one serious AE, and acute asthma attack unrelated to therapy occurred in the morphine 12 mg group. The most common TEAEs of moderate-to-severe intensity were opioid-related nausea, vomiting, dizziness, headache, pruritus, constipation, and somnolence, and these events occurred generally occurred more frequently with Q8003 12 mg/8 mg than with its individual components. No clinically relevant changes in vital signs, electrocardiogram, or clinical laboratory values were observed.

**Table 3:** Summary of TEAEs

	Number (%) of Subjects		
	
	Q8003 12 mg/8 mg (N = 171)	Morphine 12 mg (N = 176)	Oxycodone 8 mg (N = 175)
Any TEAE	152 (88.9)	111 (63.1)	122 (69.7)
Any severe TEAE	15 (8.8)	12 (6.8)	10 (5.7)
Any AE leading to discontinuation	7 (4.1)	4 (2.3)	6 (3.4)
Any SAE	0	1 (0.6)	0
Moderate-to-severe events	70 (40.9)	51 (29.0)	45 (25.7)
Nausea	36 (21.1)	31 (17.6)	23 (13.1)
Vomiting	38 (22.2)	23 (13.1)	13 (7.4)
Dizziness	10 (5.8)	5 (2.8)	7 (4.0)
Headache	8 (4.7)	17 (9.7)	5 (2.9)
Pruritus	9 (5.3)	1 (0.6)	3 (1.7)
Constipation	4 (2.3)	0	0
Somnolence	3 (1.8)	2 (1.1)	2 (1.1)

AE = adverse event; SAE = ; TEAE = treatment-emergent AE.

No episodes of respiratory rate <10/min were observed in any treatment group. The mean (SD) respiratory rate at the time of oxygen desaturation <90% was 15.1 (1.3) for Q8003 12 mg/8 mg, 15.2 (3.6) for morphine 12 mg, and 14.8 (1.1) for oxycodone 8 mg. Oxygen desaturation <90% occurred in nine subjects (27 desaturations) with Q8003 12 mg/8 mg, five subjects (11 desaturations) with morphine 12 mg, and four subjects (6 desaturations) with oxycodone 8 mg. The median duration of desaturations was 0.32, 0.54, and 0.34 hours, respectively, with Q8003, morphine, and oxycodone. Importantly, the cumulative median MED at the first desaturation was 96 mg for Q8003, 24 mg for morphine, and 42 mg for oxycodone. Of interest, 10 of the subjects (six Q8003, two morphine, and two oxycodone) with oxygen desaturation were enrolled at a study site at approximately 4,200 feet above sea level, whereas all other study sites had an elevation of <1,000 feet. The incidence of desaturation was 21.3% at this site compared with 1.9% at the five remaining study sites. Excluding the subjects from the high altitude site, the incidence of oxygen desaturation <90% was <2% in each group. The lower partial pressure of oxygen in the inspired air at higher altitude is likely to have contributed to the markedly higher desaturation rate.

## Discussion

This study enrolled subjects with moderate-to-severe pain following a unilateral bunionectomy surgery. Dosing with study medication began on the day of surgery, once the effects of local nerve blocks had sufficiently worn off. Subjects were randomized to receive either 12 mg/8 mg of Q8003 or the mg amounts of its components, 12 mg morphine or 8 mg oxycodone. The results from this study show that at twice the 12 mg MED of morphine 12 mg or oxycodone 8 mg, 24 mg MED of Q8003 (12 mg/8 mg) provided superior analgesia and satisfy the FDA requirements that a combination product demonstrate superiority (FDA Draft Guidance for Industry on Codevelopment of Two or More Investigational Drugs for Use in Combination, 2010). Q8003 was significantly superior to both comparators for SPID_24_ and SPID_48_. Q8003 also had a greater proportion of responders than either morphine or oxycodone. The 30% improvement in pain intensity scores, which was the response definition in this study, is consistent with published literature indicating that approximately a 30% reduction in pain scores on the NPRS represents a clinically important difference 19, 20. Subjects in the morphine and oxycodone groups were more than twice as likely to use rescue medication, and higher doses of rescue medication were used. This increased use of ibuprofen in the control groups relative to Q8003, may have reduced the differences in analgesic effects between 24 mg MED of Q8003 and 12 mg MED of morphine or of oxycodone. Despite administration of Q8003 at an MED that was twice that of its individual components, the incidence of TEAEs was only about 25% greater with Q8003, and importantly, 80% of the TEAEs were of mild intensity.

In this study, the effects of Q8003 on respiratory rate and oxygen desaturation were comparable with morphine and oxycodone. The duration of individual episodes of oxygen desaturation was shortest with Q8003, and the cumulative duration of episodes also was shorter with Q8003 than with morphine despite administration of twice the MED with Q8003. A potentially important aspect of the study results was the effect of high altitude of the study site on the rate of oxygen desaturation in all treatment groups compared with the other five sites located at an altitude of <1,000 feet. When subjects experiencing desaturation at clinical sites located at high altitude (at least 4,000 feet) were excluded, the desaturation rate was <2% among all three treatment groups despite a twofold higher MED with Q8003 17. This compares favorably with a 6% desaturation rate observed after a single dose of intravenous hydromorphone administered for acute pain in an emergency room setting 21.

Using preclinical pain models, dual-opioid combinations were shown to have potential benefits of enhanced efficacy, safety, and tolerability compared with single drug therapy 6. Earlier clinical work with coadministration of opioids demonstrated that analgesic efficacy was potentiated and tolerability improved in patients with postoperative 11, cancer 22, and chronic low back pain 23.

Previous studies with Q8003 have shown substantial analgesic efficacy with acceptable tolerability compared with other opioids. A randomized, double-blind, placebo-controlled study evaluated ascending doses of Q8003 in 256 hospitalized patients following bunionectomy surgery 12. Patients received placebo or Q8003 3/2 mg, 6/4 mg, 12/8 mg, and 18/12 mg for 48 hours. SPID values at 6, 24, and 48 hours were significantly (*P* < 0.05) higher than placebo with Q8003 6/4 mg, 12/8 mg, and 18/12 mg. The most common AEs with Q8003 were nausea, vomiting, pruritus, dizziness, and constipation, and while the incidence of moderate or severe nausea or vomiting was higher with Q8003 than placebo, the incidence of other AEs was comparable with placebo. The analgesic efficacy and tolerability of equi-analgesic doses of Q8003 vs monotherapy with morphine and oxycodone was evaluated in 197 subjects with moderate-to-severe pain following bunionectomy 16. Subjects were randomized to Q8003 12/8 mg or 6/4 mg, morphine 12 or 6 mg, or oxycodone 8 or 4 mg. Comparing equi-analgesic doses of Q8003 6/4 mg vs morphine 12 mg and oxycodone 8 mg, at least one AE occurred in 62.5% with Q8003 6/4 mg compared with 96.6% with morphine 12 mg and 70.6% with oxycodone 8 mg. The incidence of treatment-emergent nausea or vomiting was reduced by 50–75% with Q8003 6/4 mg compared with morphine 12 mg or oxycodone 8 mg. An open-label study compared Q8003 and oxycodone/acetaminophen (Percocet®, manufactured for Endo by Novartis, Lincoln, NE, USA) in 44 patients with acute moderate-to-severe pain following unilateral total knee arthroplasty 15. Patients were randomized to flexible-dose Q8003 12/8 mg to 24/16 mg BID, low-dose Q8003 3/2 mg to 6/4 mg BID, or oxycodone/acetaminophen 5/325 mg, one to two tablets every 4–6 hours. Flexible-dose Q8003 and the oxycodone/acetaminophen regimen exhibited comparable analgesic efficacy. However, the incidence of GI AEs was lower in the flexible-dose Q8003 group compared with the oxycodone/acetaminophen group.

The impact of opioids on side effects is important especially among patients with acute postsurgical pain. Although capable of providing effective postsurgical pain control, opioids are associated with AEs in 20% of patients, including those related to the GI system (nausea, vomiting, ileus) and CNS system (respiratory depression, confusion, delirium, etc.) and skin (pruritus) 24. Reducing these risks should be expected to lower costs associated with managing postsurgical pain. A reduction in the need for ventilatory support as well as a reduction in the duration of postoperative ileus would be beneficial in terms of decreased length of stay and, consequently, hospitalization costs 25–27.

Results from this fixed-dose study demonstrate substantial analgesic efficacy, acceptable tolerability, and no special safety concerns with Q8003 when used for the treatment of acute postoperative pain. These benefits were observed despite the use of double the MED of Q8003 compared with its individual components. Based on these results and those from previous clinical studies, Q8003 could represent an important option in the future for the treatment of acute pain.
